# *Rhodococcus equi*—Occurrence in Goats and Clinical Case Report

**DOI:** 10.3390/pathogens10091141

**Published:** 2021-09-04

**Authors:** Monika Żychska, Lucjan Witkowski, Agnieszka Klementowska, Magdalena Rzewuska, Ewelina Kwiecień, Ilona Stefańska, Michał Czopowicz, Olga Szaluś-Jordanow, Marcin Mickiewicz, Agata Moroz, Joanna Bonecka, Jarosław Kaba

**Affiliations:** 1 Division of Veterinary Epidemiology and Economics, Institute of Veterinary Medicine, Warsaw University of Life Sciences-SGGW, 02-776 Warsaw, Poland; monika_zychska@sggw.edu.pl (M.Ż.); a.klementowska13@o2.pl (A.K.); michal_czopowicz@sggw.edu.pl (M.C.); marcin_mickiewicz@sggw.edu.pl (M.M.); agata_moroz@sggw.edu.pl (A.M.); jaroslaw_kaba@sggw.edu.pl (J.K.); 2 Department of Preclinical Sciences, Institute of Veterinary Medicine, Warsaw University of Life Sciences-SGGW, 02-786 Warsaw, Poland; magdalena_rzewuska@sggw.edu.pl (M.R.); ewelina_kwiecien@sggw.edu.pl (E.K.); ilona_stefanska@sggw.edu.pl (I.S.); 3 Department of Small Animal Diseases with Clinic, Institute of Veterinary Medicine, Warsaw University of Life Sciences-SGGW, 02-776 Warsaw, Poland; olga_szalus_jordanow@sggw.edu.pl (O.S.-J.); joanna_bonecka@sggw.edu.pl (J.B.)

**Keywords:** rhodococcosis, ruminants, abscesses

## Abstract

Background: *Rhodococcus equi* infection is commonly known in equine medicine to cause frequently fatal rhodococcosis. Infections in other species and people are also reported. Clinical manifestation in goats is relatively similar to horses and humans, but data regarding bacterium prevalence are scarce. Thus, the study aimed to estimate the occurrence of *R. equi* in goats. Methods: During post mortem examination, submandibular, mediastinal, and mesenteric lymph nodes were collected. Standard methods were used for bacteria isolation and identification. Results: A total of 134 goats were examined, and 272 lymph node samples were collected. *R. equi* was isolated from four animals. All four isolates carried the *choE* gene, and one also had *traA* and pVAPN plasmid genes. Conclusions: To the authors’ best knowledge, this is the first report of *R. equi* occurrence and genetic diversity in goats. The results may help create a model for treating rhodococcosis in other animal species and assessing the role of meat contamination as a potential source of human infection. This research should be considered a pilot study for further application of the goat as a model of *R. equi* infection in horses and humans.

## 1. Introduction

*Rhodococcus equi* is a ubiquitous bacterium. The genus *Rhodococcus* is closely related to the *Mycobacterium* and *Corynebacterium* genera. Bacteria of this genus are described as aerobic, Gram-positive coccobacillus-invading macrophages. *Rhodococcus equi* is widely known as a causative agent of purulent bronchopneumonia in foals–rhodococcosis. The disease primarily affects foals in the first three months of life. Due to its high morbidity and mortality and the costs associated with treatment, it has a significant financial impact on the horse industry worldwide. However, our understanding of the disease is limited. Rhodococcosis in foals still presents a clinical and scientific challenge, and many aspects remain unclear [1,2,3,4,5,6].

The combination of a macrolide and rifampicin has been the mainstay of rhodococcosis therapy in foals for decades, and now, increasing antimicrobial resistance is a growing threat, extending beyond equine medicine. *R. equi* affects other animals and is a human zoonotic pathogen, and these drugs are widely used in humans, for example, to treat tuberculosis [1,2,3,4,5,6,7,8].

Even though *R. equi* is not considered an essential threat to species other than horses, a growing number of reports of *R. equi* infection in farm and wild animals, including cattle, goats, sheep, lamas, camels, buffaloes, roe deer, deer, pigs, wild boars, and wild birds have been published in recent years [9,10,11,12,13,14,15,16,17,18,19,20].

The first reports of *R. equi* infection in goats come from 1974 from India and the USA [21], and only a dozen articles have been published on this topic [15,17,18,22,23,24,25,26,27,28,29]. However, recent reports of infections in companion animals appear to be more thrilling in the possible transmission and even faster increase of antimicrobial resistance. Thus, this tendency is likely to change [9,11,30,31,32,33,34,35,36].

Even though the literature on *R. equi* infection in animals—especially foals—is extensive, the amount of data regarding disease importance in human medicine is somewhat limited. However, in the last few decades, a growing number of cases has been reported, and *R. equi* has recently gained attention as an opportunistic pathogen in human beings. The first case dates to 1967: a 29-year-old man with autoimmune hepatitis in chronic steroid therapy—*R. equi* was isolated from the lung and subcutaneous abscess [37]. However, *R. equi* became more widely recognized with the onset of the human immunodeficiency virus (HIV)/AIDS epidemic. In 1986, *R. equi* was first detected in a patient with AIDS [38]. Later, due to the increasing number of HIV infections, the development of transplantation, and diagnostic methods, the number of diagnosed cases of *R. equi* surged in apparently immunocompetent individuals. Nowadays, the amount of infection in immunocompromised people is growing, which is highly alarming due to the emerging antibiotic resistance of *R. equi* [8,33,38,39,40,41,42,43,44,45,46].

To date, the source of *R. equi* infection for humans remains unknown. However, it became apparent that contact with the horses (or their environment) is not the only factor in human disease. Genetic studies have shown more frequent isolation of pig- and cattle-specific strains than equine or environmental strains. Therefore, human infection via meat consumption is the most probable. pVAPB strains specific to pigs and wild boars were isolated from humans who usually did not directly come in contact with those animals and their environment [16,36,47]. The bovine-specific pVAPN strains were detected in the lymph nodes of cattle and goats and the lungs of HIV patients [23,46,48]. However, clinical cases resulting from contact with damaged skin/mucous membranes and contaminated soil, human-to-human transmission, and hospital infections are also described [8].

*R. equi* infection in foals mainly causes pyogranulomatous bronchopneumonia, but extrapulmonary disorders (EPDs) have also been observed [1,2,3,4,5,6]. In cattle, pigs, and wild boars, *R. equi* infection is mainly associated with tuberculous-like lesions in lymph nodes [12,16,36,47,48,49,50,51,52]. Disseminated organ abscessation with frequent involvement of the liver and lungs, as well as concurrent lymphadenitis and osteomyelitis, are most often reported in infected goats [15,17,18,21,22,23,24,25,26,27,28,29]. The cutaneous and pulmonary form of the disease is reported in cats and dogs [9,11,30,31]. Pneumonia is the most common manifestation in humans, but EPDs, including pericarditis, mastitis, empyema, pericarditis, mediastinal and intra-abdominal lymphadenopathy, brain and psoas abscesses, osteomyelitis, and spondylodiscitis are also observed. There are also cases of sepsis in preterm infants who had respiratory distress [8,43,44,45].

Given the similarities mentioned above to the disease course in foals, we decided to estimate the occurrence of *R. equi* in the lymph nodes of goats.

## 2. Results

**Figure 1:**
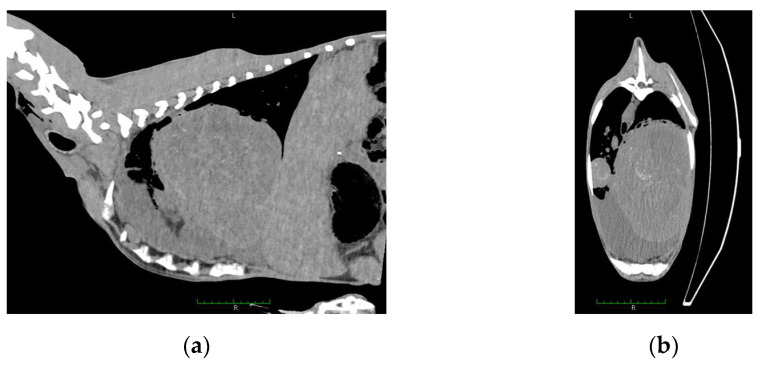
Computed tomography image of the goat: (**a**) sagittal section, abscess (19 cm length, 20 cm high) located in the thorax, (**b**) frontal section, abscess (17 cm) located in the thorax. Both confirmed by *R. equi* isolation.

**Figure 2:**
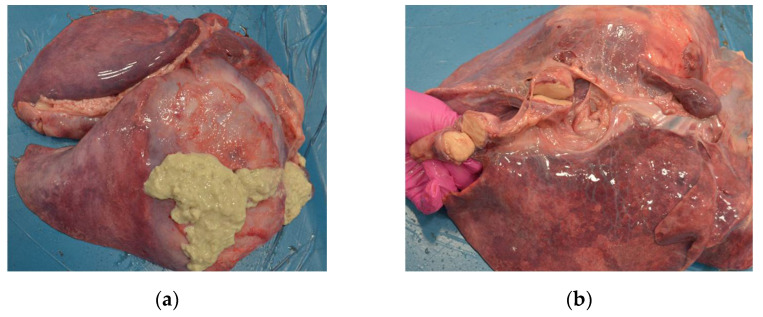
Necropsy lesions of the goat caused by *R. equi*: (**a**) lungs abscessation, (**b**) abscesses in the mediastinal lymph nodes.

**Figure 3:**
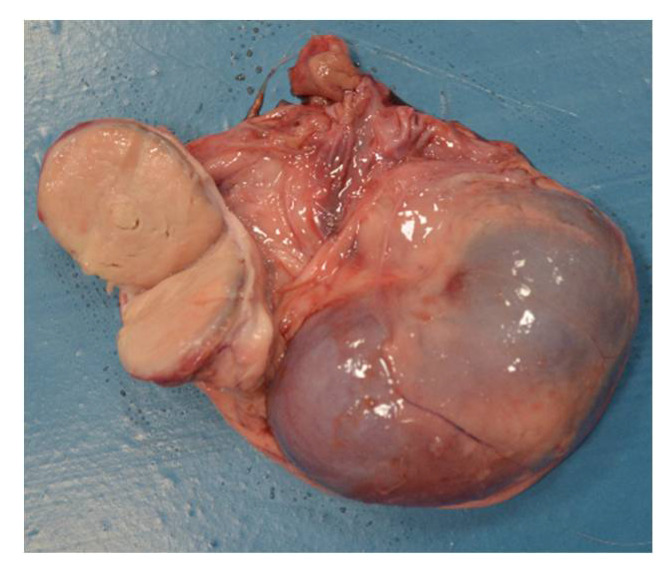
Necropsy lesions of the goat: kidney abscessation caused by *R. equi*.

**Figure 4:**
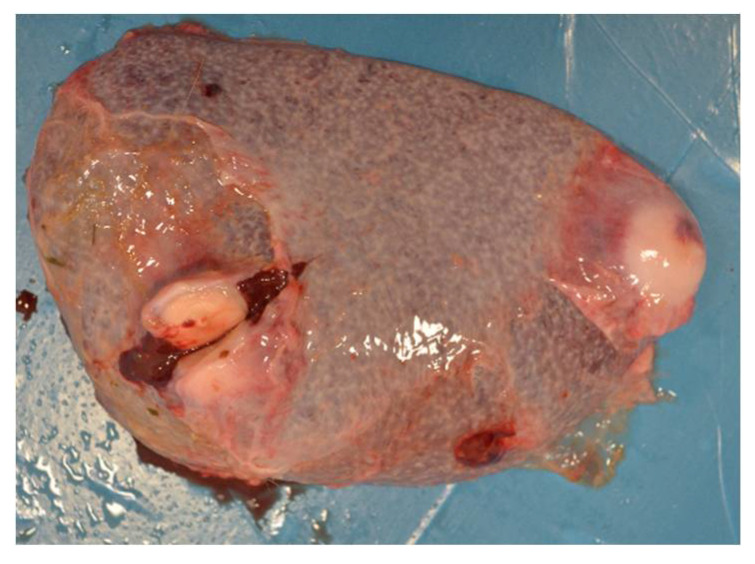
Necropsy lesions of the goat: spleen abscessation caused by *R. equi*.

**Figure 5:**
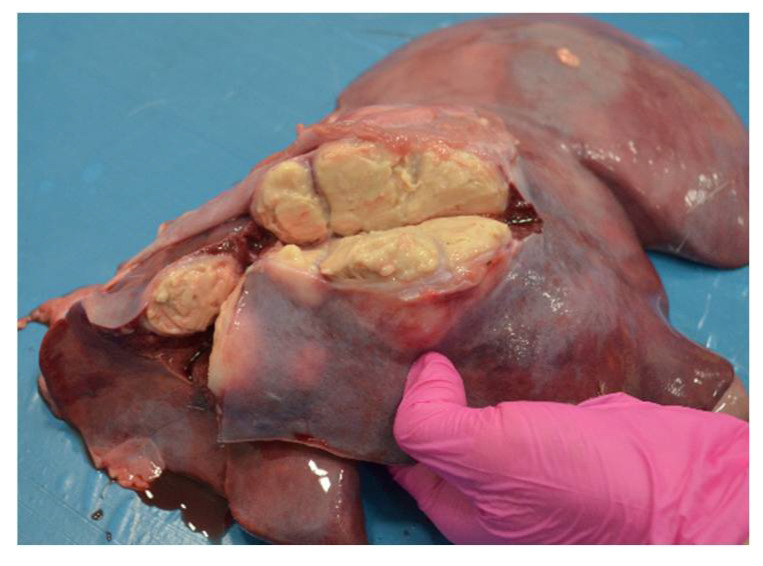
Necropsy lesions of the goat: liver abscessation caused by *R. equi*.

A total of 134 goats were examined. Computed tomography showed multiple lesions in the thorax and abdomen in one animal (0.76%) (Figure 1). During necropsy, disseminated abscesses in the lungs (Figure 2), kidneys (Figure 3), spleen (Figure 4), liver (Figure 5), and mesentery were found. *R. equi* was isolated from all investigated lesions, mostly in pure culture, but in some lesions, the co-infection with *Corynebacterium pseudotuberculosis* and *Trueperella pyogenes* was confirmed.

In two animals, purulent lesions were present in collected lymph nodes (both in the mediastinal lymph nodes). In addition, extra-lymphatic abscesses were observed in some of the animals, and from most of the lesions, *Corynebacterium pseudotuberculosis* and *Trueperella pyogenes* were isolated.

From all 134 investigated animals, 272 lymph node samples were collected: 122 submandibular lymph nodes, 61 mediastinal lymph nodes, and 89 mesenteric lymph nodes.

*R. equi* was isolated from four animals: in three cases, from lymph node samples (two mesenteric and one submandibular) without any lesions; and the fourth isolate came from a goat suffering multiple organ abscessation. All four isolates were Gram-positive coccobacilli, and their growth characteristics were typical for the *R. equi* (mucoid, salmon-pink on blood agar, and greyish on CAZ-NB medium, irregular). They were catalase-positive, oxidase-negative, and identified to the genus level as *Rhodococcus* by API Coryne. In the CAMP test, enhancement of hemolysis was observed in the presence of both indicatory bacterial strains for these two isolates. In addition, all four isolates carried the *choE* gene specific to the species.

Unfortunately, two of four *R. equi* isolates, one from the lymph node and an isolate from the goat with multiple organ abscessation because of technical issues, were not available for further investigation.

One of the two remaining isolates carried *traA* and *vapN* genes. None of the plasmid-associated virulence genes were found in the second caprine *R. equi* isolate.

*Corynebacterium pseudotuberculosis* and *Trueperella pyogenes* were detected in two and one lymph node samples, respectively. In addition, *Staphylococcus* spp. was detected in the lymph nodes of nine animals (Table 1).

PCR product obtained for the *vapN* gene was sequenced (outsourced to the Genomed, Poland) and analyzed with the Basic Local Alignment Search Tool (BLAST) carried out on the National Center for Biotechnology Information (NCBI) website (http://blast.ncbi.nlm.nih.gov accessed on 8 January 2020). As a result, the *vapN* gene sequence was deposited in the GenBank database under the accession number MN913373.1.

## 3. Discussion

This study continues the research on *R. equi* occurrence in farm animals led by the authors. However, in this case, the outcome may be used not only as epidemiological data, but may also impact further studies on *R. equi* pathogenicity.

According to the authors’ knowledge, this kind of study, designed to investigate the occurrence of *R. equi* in goats, was performed for the first time. So far, clinical cases were published [15,17,18,21,22,23,24,25,26,27,28,29], but only one study on the occurrence of *R. equi* in goats was performed. The serological screening in one breeding farm shoved seroprevalence close to 30% and confirmed exposure of clinically healthy goats for vapN-harboring *R. equi* [23]. Thus, it is not possible to compare the results with other goat studies.

Generally, ruminants are considered relatively resistant to *R. equi* infection. Thus, detection of the single clinical case between 134 investigated goats is not surprising. Additionally, detection of *R. equi* in 1.1% of investigated goats’ lymph nodes is close to other studies on ruminants. Prevalence of *R. equi* in slaughtered cattle used to be considered very low (0.008%) [53], but more recently, *R. equi* was isolated from 1.3% of lesion-free lymph nodes of cattle carcasses approved for human consumption [16]. Furthermore, the low prevalence of avirulent, environmental strains of *R. equi* in red deer (0.7%) and roe deer (0.9%) and lack of tissue lesions indicate an accidental carriage of the pathogen [36]. In addition, isolation of the ruminant-specific (pVAPN-carrying) *R. equi* aligns with previous studies. It confirms that the goat’s environment or goat meat might be a potential source of infection for humans [23,46,54].

Reported *R. equi* detection in cattle primarily concerns animals with purulent lesions or those suspected of *Mycobacterium* spp. infection similarly to the American bison with paratuberculosis [50,51,52]. On the other hand, microbiological examination of purulent lesions and caseous lymphadenitis found in slaughtered sheep during meat inspection detected some bacteria species, but was negative for *R. equi* [55].

This study has some limitations. Because the material was collected during another project carried out by the same research group, it was impossible to obtain all needed samples from each animal. In some cases, other pathological lesions precluded the collection of the lymph nodes for this survey. Furthermore, the collected material was stored at −20 °C until testing, which could reduce the effectiveness of *R. equi* isolation, so the accurate scale of the problem may be considerably more significant. However, the same methodology was fully effective in previous studies on the epidemiology of *R. equi* infection in wild and slaughter animals [16,36] and detection of *Corynebacterium pseudotuberculosis*, *Trueperella pyogenes,* and *Staphylococcus* spp., the most often isolated pathogens from purulent lesions in goats, confirms the usage of appropriate methods [56,57,58,59].

Moreover, samples were collected only from goats from one herd, and the investigated animals were not healthy. Furthermore, individuals were not randomly selected, but eliminated from the herd because of advanced caprine arthritis-encephalitis (CAE) clinical findings and weak conditions. This issue may pose a problem, as this comorbidity may affect the immunological status of the animal and therefore be unrepresentative of the rest of the population. However, CAE infection is widespread in Poland (over 80% of goat herds), and seroprevalence in the herds can approach 100% of adult animals [60,61,62]. Therefore, animals should not be excluded due to CAE infection, as they may represent a considerable part of the goat population.

It is believed that factors predisposing goats to *R. equi* infection are all immune-suppressing factors, such as stress (transport), co-existing diseases (parasites), and poor environmental conditions that have been published [15,17,18]. The direct impact of the small ruminant lentivirus (SRLV) infection on goats’ immunological status is not known. However, animals with advanced CAE clinical findings are under permanent stress deriving from the illness, for example, pain, the inability to move, and limited access to food and water. In addition, the social behavior of the herd might also have an impact because ill individuals are in the lowest position in the herd hierarchy. All those factors may lead to a weaker immunological response.

Thus, goats’ risk factors and clinical outcomes are similar to those observed in humans, implying that findings of goats suffering from severe CAE might reflect on immunocompromised humans. Therefore, undertaking research using goats as a large animal model may further develop a better understanding of the disease.

## 4. Materials and Methods

The study was performed on Polish White Improved and Polish Fawn Improved goats. Animals enrolled in this study were eliminated from a large dairy herd due to severe caprine arthritis-encephalitis (CAE) clinical findings, mainly due to emaciation, low milk yield, or progressive arthritis.

The culled animals were used for several research projects. Among others, computed tomography (CT) and necropsy were performed [63,64]. During post mortem examination, material for various laboratory procedures, including swabs from abscesses, was obtained. Moreover, submandibular, mediastinal, and mesenteric lymph nodes were collected and stored at −20 °C until testing.

Standard methods were used for bacteria isolation and identification. Briefly, lymph nodes were initially crushed with sterile scissors, and then 1 g of tissue was homogenized in 3 mL of 0.9% saline solution using a PRO200 homogenizer Multi-Gen 7 (PRO Scientific Inc., Oxford, CT, USA). Next, 100 µL of homogenate was collected and cultured on plates with a differentiation medium of Columbia Agar supplemented with 5% sheep blood (Graso Biotech, Starogard Gdanski, Poland), and selective CAZ-NB medium (Mueller-Hinton agar base supplemented with ceftazidime (0 µg/mL) and novobiocin (25 µg/mL) modified by the addition of 0.026% cycloheximide and 0.005% potassium tellurite. Plates were incubated for 48 h at 37 °C in aerobic conditions [16,36,49].

Colonies were identified based on their morphological, cultural, and biochemical characteristics. Gram staining was used to determine the cell morphology of isolates. Additionally, CAMP with *Staphylococcus aureus* ATCC 25923 with the *R. equi* ATCC 33701 reference strain as the control was performed on Columbia Agar with a 5% addition of sheep blood. The test results were evaluated after 24 h of incubation at 37 °C under aerobic conditions. The biochemical properties of isolates were tested using the API Coryne test (bioMérieux, Marcy l’Etoile, France) according to the manufacturer’s instructions.

DNA extracted from cultures of *R. equi* isolates that were 24 hours old was used as a template for PCR. Each isolate colony was suspended in 500 µL of distilled water and incubated at 99 °C for 10 min. Then, samples were cooled on ice and centrifuged, and stored at −20 °C. The presence of five *R. equi* genes, *choE*, *traA*, *vapA*, *vapB*, and *vapN*, was determined by PCR using specific primers (Table 2). The reaction mixture with a final volume of 25 µL contained 9.5 µL of nuclease-free water (Thermo Scientific, Waltham, MT, USA), 12.5 µL of DreamTaq Green PCR Master Mix (Thermo Scientific, Waltham, MT, USA), 10 pmol of each primer (Genomed, Warsaw, Poland), and 1 µL of DNA template. The thermal cycling conditions for the *choE*, *traA*, *vapA*, *vapB*, and *vapN* genes were conducted as previously described [9,16,37,65]. The amplified PCR products were separated by electrophoresis through 1% agarose gel in TAE buffer stained with Midori Green DNA Stain (Nippon, Düren, Germany), visualized, and analyzed using a VersaDoc Model 1000 Imaging System and Quantity One software (version 4.4.0) (Bio-Rad, Hercules, CA, USA).

## 5. Conclusions

Despite its limitations, the study results show that *R. equi* is present in the goat population. Therefore, goat meat consumption is a possible source of human infection. It might also be speculated that the goat might be reconsidered as an inexpensive and applicable large animal model for *R. equi* infection.

Further research on the occurrence and pathogenicity of *R. equi* in goats might create a possibility for better understanding of the disease and development of the model, enabling the introduction of novel diagnostics and treatment techniques.

## Figures and Tables

**Table 1:** Result of the microbiological examination of the lesion-free lymph nodes collected from the goats.

Bacterium	No. of Affected Goats	No. (%) of Lymph Nodes from Which the Bacterium Was Isolated		
		Mesenteric (n = 89)	Submandibular (n = 122)	Mediastinal (n = 61)
*Rhodococcus equi*	3	2	1	0
		(2.2)	(0.8)	(0.0)
*Corynebacterium pseudotuberculosis*	2	0	1	2
		(0.0)	(0.8)	(3.3)
*Staphylococcus* spp.	9	8	2	4
		(9.0)	(1.6)	(6.6)
*Trueperella* spp.	1	1	0	1
		(1.1)	(0.0)	(1.6)
others	2	1	2	0
		(1.1)	(1.6)	(0.0)

**Table 2:** PCR primers used to detect the selected genes of *R. equi*.

Target Gene	Gene Product	Primer Sequence (5′–3′)	Amplicon Size	Reference
*choE*	cholesterol oxidase	F-GTCAACAACATCGACCAGGCG	959 bp	[66]
		R-CGAGCCGTCCACGACGTACAG		
*traA*	protein of the conjugal transfer machinery	F-AGAGTTCATGCGTGACAACG	959 bp	[67]
		R-GTCCACAGGTCACCGTTCTT		
*vapA*	virulence-associated protein A	F-GACTCTTCACAAGACGGT	564 bp	[65]
		R-TAGGCGTTGTGCCAGCTA		
*vapB*	virulence-associated protein B	F-TGATGAAGGCTCTTCATAA	589 bp	[68]
		R-TTATGCAACCTCCCAGTTG		
*vapN*	virulence-associated protein N	F-GCACTCCAAAAATACCCCGGAAG	625 bp	[9]
		R-CTTTGCCAGGTCTTGCGAATGTTAT		

## Data Availability

GenBank database under the accession number MN913373.1.
